# Molecularly Imprinted Membrane Produced by Electrospinning for β-Caryophyllene Extraction

**DOI:** 10.3390/ma15207275

**Published:** 2022-10-18

**Authors:** João de Deus Pereira de Moraes Segundo, Maria Oneide Silva de Moraes, Walter Ricardo Brito, Robert S. Matos, Marco Salerno, Yonny Romaguera Barcelay, Karen Segala, Henrique Duarte da Fonseca Filho, Marcos Akira d’Ávila

**Affiliations:** 1 Department of Manufacturing and Materials Engineering, University of Campinas, Campinas 13083-860, Brazil; 2 Department of Chemistry, Federal University of Amazonas, Manaus 69067-005, Brazil; 3 Thematic Laboratory of Microscopy and Nanotechnology, National Institute of Amazonian Research, Manaus 69067-001, Brazil; 4 Postgraduate Program in Materials Science and Engineering, Federal University of Sergipe-UFS, São Cristóvão 49100-000, Brazil; 5 Institute for Globally Distributed Open Research and Education (IGDORE), Institute for Materials Science, Max Bergmann Center of Biomaterials, Technische Universität Dresden, 01069 Dresden, Germany; 6 BioMark@UC/CEB–LABBELS, Faculty of Sciences and Technology, University of Coimbra, 3004-531 Coimbra, Portugal; 7 Laboratory of Synthesis of Nanomaterials and Nanoscopy, Physics Department, Federal University of Amazonas-UFAM, Manaus 69067-005, Brazil

**Keywords:** molecular printing technology, electrospinning, molecularly imprinted membrane, β-caryophyllene, organic compounds

## Abstract

Molecularly imprinted membrane of β-caryophyllene (MIM–βCP) was fabricated incorporating β-caryophyllene molecularly imprinted polymer nanoparticles (βCP–NP) into polycaprolactone (PCL) fibers via electrospinning. The βCP–NP were synthesized by precipitation polymerization using the βCP as a template molecule and acrylic acid as a functional monomer in the proportion of 1:4 mol, respectively. Atomic force microscopy images and X-ray diffraction confirmed the nanoparticles’ incorporation into MIM–βCP. MIM–βCP functionalization was evaluated by gas chromatography. The binding capacity was 1.80 ± 0.05 μmol/cm^2^, and the selectivity test was performed with a mixing solution of βCP and caryophyllene oxide, as an analog compound, that extracted 77% of the βCP in 5 min. The electrospun MIM–βCP can be used to detect and extract the βCP, applications in the molecular sieve, and biosensor production and may also contribute as an initial methodology to enhance versatile applications in the future, such as in the treatment of skin diseases, filters for extraction, and detection of βCP to prevent counterfeiting of commercial products, and smart clothing with insect-repellent properties.

## 1. Introduction

Molecular printing technology has been widely used to obtain molecularly imprinted polymers (MIPs) with selectivity and ability to recognize specific analytes [1,2]. MIPs have a wide range of applications in important areas, such as in chemical, optical, and biological sensors, protein selection, pharmaceutical analysis, and chromatography [3,4]. The main chemical components of MIPs are the functional monomer, the template, the cross-linking agent, the initiator, and the solvent [5]. Several methods are used to obtain MIPs, and, depending on their application, it is possible to obtain them in different physical forms [6]. These methods generally occur through the polymerization mechanism by free radicals, which include mass polymerizations, suspension, emulsion, precipitation, and others [1,6].

MIPs are generally applied in the form of particles. For this reason, nanometric dimensions are desirable due to the high surface area [7]. Moreover, new materials can be developed from MIP nanoparticles [1,6]. Attractive alternatives have been reported in the literature to develop MIPs for specific applications, such as molecularly imprinted membranes (MIMs) [8,9,10], MIP nanofiber membranes by electrospinning [7,11,12,13], nanostructured MIPs [14], MIP scaffold [15,16] and other forms, such as films and magnetic nanoparticles of molecular printing [17].

Electrospun MIMs can be obtained by combining solution electrospinning and molecular printing by dispersing MIP nanoparticles into electrospinable polymeric solutions. A strong electric field stretches the solution towards the grounded metal collector. In this path, the solvent evaporates, and the overlapping MIP nanoparticles-loaded electrospun nanofibers are deposited on the grounded metallic collector [11,18,19,20].

MIP particles of rhodamine B (Rh-B) were synthesized by the precipitation method and incorporated into polyethylene terephthalate (PET) fibers via electrospinning; the molecular printing technology and electrospinning technique showed themselves appropriate to develop Rh-B MIM that exhibits selective adsorption of the molecule [7]. Moreover, molecularly imprinted polystyrene (PS) nanofibers were fabricated by electrospinning, resulting in excellent pesticide adsorption [21]. With a distinct method, using electrospinning, Xue et al. [22] fabricated a MIM of 2,4-dinitrotoluene (MIM-DNT). However, the authors combined PET with a functional macromer as the polymeric matrix and DNT as the template molecule. The MIM-DNT was immersed in a DNT aqueous solution of known concentration and extracted and separated the DNT from the solution.

βCRF is a natural substance of a vegetal source known as caryophyllene [23]. In general, the βCRF is present in dietary ingredients, e.g., basil (*Ocimum* spp.), cinnamon (*Cinnamomum zeylanicum*), clove (*Syzygium aromaticum*), hops (*Humulus lupulus*), lavender (*Lavandula angustifolia*), oregano (*Origanum vulgare*), black pepper (*Piper nigrum*), and rosemary (*Rosmarinus officinalis*) [24]. βCP has the status of being generally recognized as safe (GRAS), as assigned by the food and drug administration (FDA) [24]. The main applications of βCP are in the pharmaceutical and cosmetic areas due to its excellent antioxidant, anti-inflammatory, antiviral, anticancer, and antifungal properties [23,25].

MIP-βCP has been synthesized by mass polymerization. MIP-βCP was used to produce a new electrochemical sensor to detect βCP; this molecule has excellent anti-inflammatory properties and can be found in plant extracts, oils, and resins [23]. On the other hand, PCL is a hydrophobic polymer [26], semicrystalline aliphatic polyester, and has good mechanical properties. PCL is suitable for electrospinning and has been extensively used to fabricate fibers incorporating nanoparticles for several applications [14]. Moraes Segundo et al. [27] obtained βCP–NP and incorporated them into PCL fibers by electrospinning. The material is promising in the pharmaceutical area, analytical analysis, biosensors, and biomedicine. In addition, it may contribute to versatile applications in the future, such as tissue engineering in treating skin diseases, filters for extracting and detecting βCP to prevent counterfeiting of commercial βCP-based products, and smart clothing with the properties of insect repellent.

In this work, we applied scanning electron microscopy (SEM) and atomic force microscopy (AFM) to characterize the PCL fibers and incorporation of βCP–NP in those fibers. From the AFM topographic images, several stereometric parameters can be obtained through advanced analysis, which have been used recently to characterize chemical and biological surfaces, such as biofilms [28,29], leaves [30], teeth [31], and wood [32]. These parameters can provide important information on the physical and functional properties of the surface, such as flatness, texture symmetry, roughness, shape of rough peaks, texture direction, and density. The morphological analysis aiming to identify the incorporation of βCP–NP into the PCL fibers was supported with chemical and structural information provided by gas chromatography coupled to mass spectroscopy (GC-MS) and X-ray diffraction (XRD) measurements, respectively.

## 2. Materials and Methods

### 2.1. Materials

Ethyleneglycoldimethacrylate (EGDMA, 98%), 2,2′-Azobis (2-methylpropionitrile) (AIBN, 98%), acetonitrile (ACN, 99.8%), acrylic acid (AA, 99%), βCP (MM = 204.35 g/mol), caryophyllene oxide (CP–oxide, MM = 220.35 g/mol), chloroform (99.5%), and PCL (MM = 70,000–90,000 g/mol) were all supplied by Sigma Aldrich. Acetone (99.5%) was supplied by Synth and methanol (99.8%) by Neon. Deionized water was used, with electrical conductivity of 0.5 μS/cm^2^.

### 2.2. Synthesis of βCP–NP and Production of Electrospun MIM–βCP

The βCP–NP synthesis and the MIM–βCP fabrication were carried out following the description by Moraes Segundo et al. [27]. The βCP as a template molecule and AA as a functional monomer were mixed into the flask in the 1:4 mol/mol ratio. Next, 30 mL of acetonitrile, 10 mmol of EGDMA, and 0.25 mL de AIBN were added into the flask and taken to an ultrasonic bath for 15 min. The polymerization solution was purged with N_2_ gas for 5 min. Then, it was heated at T = 60 °C under mechanical stirring at 1000 rpm. The polymeric reaction lasted 24 h. Finally, the βCP–NP was obtained after centrifugation at 5000 rpm and stocked for use.

To prepare the MIM–βCP, the PCL solution was obtained by dissolving 1 g PCL in 7.2 mL of chloroform and acetone mixture in the ratio 1:1 *wt*/*wt* under mechanical stirring for 4 h at room temperature. βCP–NP (30 mg) was added into PCL solution under mechanical stirring for 1 h. Next, the electrospinning solution was transferred into a syringe. Electrospinning-setup parameters, such as needle diameter, flow rate, tension, and work distance, were 0.8 mm, 8 mL/h, 15 kV, and 180 mm, respectively. The speed of the rotating cylinder device was 450 rpm. The MIM–βCP was fabricated by deposition overlapping βCP–NP-loaded PCL fibers on the grounded rotating cylinder. The non-imprinted membrane (NIM) followed the same preparation methodology as described previously, but without the presence of βCP, and was used to compare with the functionalized MIM–βCP in the βCP extraction test and binding capacity.

### 2.3. Analysis of βCP–NP by DLS

βCP–NP size and distribution were investigated by dynamic light scattering (DLS) using a particle size analyzer (Malvern Instruments, Mastersizer 3000, Malvern, UK) with scattering angle of 90°, temperature of 25 °C, and viscosity of the dispersion medium of 0.893 mPa s.

### 2.4. Morphological Characterization by SEM

Synthetized βCP–NP, electrospun PCL mat, MIM–βCP, functionalized MIM–βCP, and NIM were observed using a SEM Evo MA 15 (Zeiss, Oberkochen, Germany). The samples were metalized with 10 nm coating of gold using a sputter coater CPD 050 (BAL TEC, Balzers, Liechtenstein). Fiber diameters were measured according to the method described in the literature [27,33].

### 2.5. Analysis of βCP–NP Incorporation into PCL-Fibers

#### 2.5.1. AFM Analysis

Atomic force microscopy was used to investigate the surface morphology of the electrospun PCL fiber and βCP–NP-loaded PCL fibers. The samples were prepared according to the method proposed by the Backer et al. [34]. A piece of glass slide with double-sided adhesive tape was placed on the rotating collector surface. The electrospun PCL fibers with and without βCP–NP were deposited on the glass slide at time intervals of 5 to 10 s. To perform the AFM, the samples were fixed on a sample holder using a double-sided adhesive tape, and the measurements were carried out at room temperature (25 °C) and 60 ± 1% relative humidity on an AFM Innova (Bruker, Billerica, MA, USA). The surfaces were scanned in tapping mode, using a silicon tip with Al coated cantilever, having a nominal spring constant of 42 N/m, at a scan rate of 0.5 Hz and scan areas of 2 × 2 µm^2^ and 450 × 450 nm^2^, with 256 × 256 pixels. All AFM images were analyzed using Mountains Map^®^ Premium software trial version 8.4.8872. The stereometric parameters were extracted according to ISO 25178-2: 2012 [35], and their meaning was assumed as presented in several other works [28,36,37].

#### 2.5.2. Statistical Analysis

The statistical analysis was carried out using the OriginPro^®^ software version 8.0724 (OriginLab Corporation, Northampton, MA, USA). One-way Analysis of Variance (ANOVA) pair comparisons was performed with a Tukey test at a *p*-value of 0.05.

#### 2.5.3. XRD Spectroscopic Characterization

The PCL fiber and βCP–NP-loaded PCL fibers were analyzed by X-ray diffractometer XRD 6000 (Shimadzu, Kyoto, Japan) equipped with a graphite monochromator and Cu anode ceramic X-ray tube, radiation from the Kα1 and Kα2 lines of wavelengths, λ = 0.154056 and 0.154439 nm, respectively, with 30 mA current and 45 kV voltage. Measurements were performed in the angular range from 5 to 65° (2θ).

The degree of crystallinity (X_c_) of PCL fibers and βCP–NP-loaded PCL fibers was calculated at the crystalline and non-crystalline areas using the equation:

Xc = I_c_/(I_c_ + I_a_) (1)

where I_c_ is the area of crystalline peaks, and I_a_ is the area of non-crystalline peaks [38].

### 2.6. Analysis by Gas Chromatography

GC-MS was carried out on an instrument Trace GC Ultra (Thermo Scientific, Waltham, MA, USA), operated with an inert gas at flow rate of 20 mL/min, split mode (1:20 ratio). The column temperature was 100 to 250 °C at a rate of 5 °C/min, and methanol was used as the solvent. βCP and CP–oxide were identified by mass spectra and retention time [39]. Below, all experimental procedures of the functionalization process, calibration curve, βCP extraction, and selectivity test were performed using the GC-MS.

**Figure 1:**
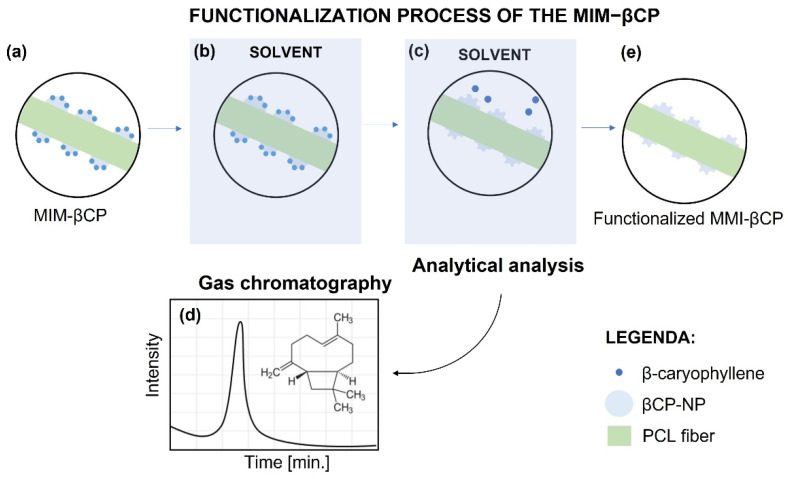
Experimental scheme of the functionalization process of MIM–βCP. (**a**) Electrospun MIM–βCP. (**b**) Electrospun MIM–βCP immersed into the solvent. (**c**) Removal of βCP from MIM–βCP. (**d**) Chromatogram of the βCP removed by wash. (**e**) Functionalized MIM–βCP.

The functionalization process of electrospun MIM–βCP was performed by washing in methanol to remove the βCP from the MIM–βCP structure. The MIM–βCP (64 mg with an area of 9 cm^2^) was immersed in a glass flask containing 5 mL of methanol and conducted to the ultrasound bath for 5 min. Next, it was removed and immersed in another glass flask containing 5 mL of methanol and subjected to an ultrasound bath. The washing sequence was repeated four times. An aliquot of 2 mL of each wash was collected and analyzed by GC-MS at a retention time of 9.48 min. Figure 1 briefly shows the steps taken to obtain the aliquots that were analyzed in the GC-MS of functionalization process of the MIM–βCP.

The calibration curve was obtained preparing standard solutions of βCP in methanol with 0.2, 0.4, 0.6, 0.8, 1.0, and 1.2 mM. The solutions were analyzed in the GC-MS and its chromatograms obtained. Next, the peak areas were measured with the Xcalibur software and plotted versus standard concentrations. Subsequently, the calibration curve was obtained according to the following equation:

y = a x [C] + b(2)

where y is the peak area of the chromatogram, C is the concentration of βCP, and a and b are constants. The CP–oxide calibration curve was obtained following the same procedure.

The βCP extraction was performed using the functionalized MIM–βCP that was immersed into a 25 mL βCP methanolic solution of 1.2 mM concentration. The chromatograms were monitored at times of 0, 5, 10, 15, and 20 min. The concentrations were measured using the βCP calibration curve and inserted into the following equation to calculate the βCP extraction:

βCP extraction = ((C_i_ − C_f_)/C_i_) × 100 [%] (3)

where C_i_ and C_f_ are the initial and final concentrations (mM), respectively. All the measurements were obtained in triplicate.

The binding capacity of functionalized MIM–βCP was obtained by its immersion into βCP solutions (25 mL) with different concentrations of 0.2, 0.4, 0.6, 0.8, 1.0, and, 1.2 mM, at the time that was previously determined as 5 min. An aliquot of 2 mL was removed at each experimental step and analyzed by GC-MS. The chromatograms were obtained, and the concentrations were measured by a βCP calibration curve and inserted in the following equation to calculate the binding capacity in µmol/cm^2^:

Q_MIM_ = ((C_i_ − C_f_)/A) × V (4)

where C_i_ and C_f_ are the initial and final concentrations (mM), V is the volume, and A is the area of functionalized MIM–βCP. All the measurements were obtained in triplicate.

The selectivity test of functionalized MIM–βCP was performed using CP–oxide as an analog compound. Methanolic solutions of βCP/CP–oxide mixtures (20 mL) were prepared in the proportions of 30:70, 50:50, and 70:30 *vol*/*vol*, respectively. The functionalized MIM–βCP was immersed into a mixture solution, and a 2 mL aliquot was collected at 0, 5, 10, 15, and 20 min and analyzed by GC-MS.

## 3. Results and Discussion

### 3.1. Morphology of βCP–NP and Electrospun MIM–βCP

**Figure 2:**
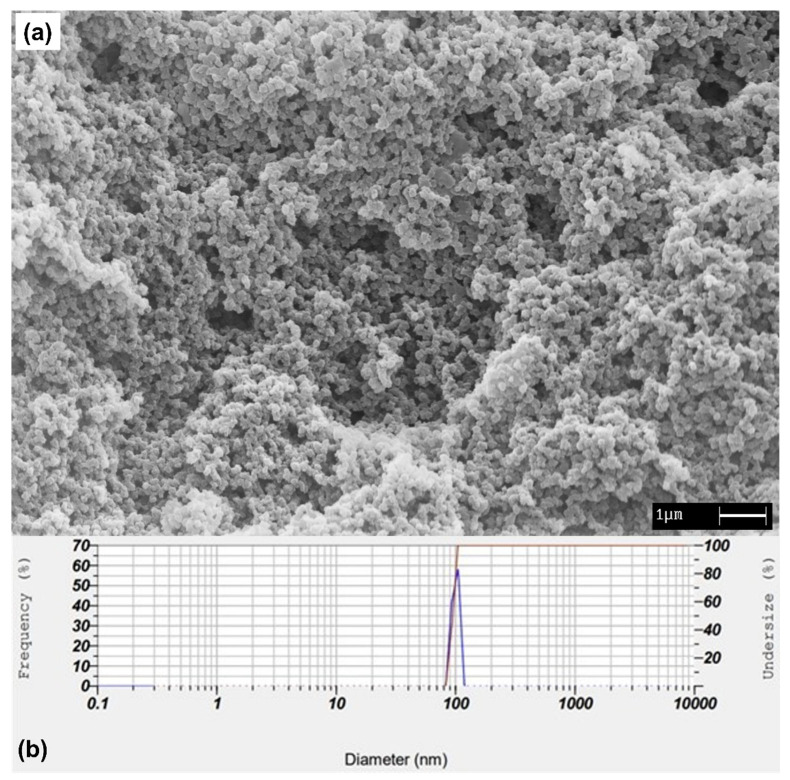
(**a**) SEM image (20,000×) and (**b**) DLS size distribution of the synthetized βCP–NP obtained by precipitation polymerization.

The morphology of βCP–NP synthesized by precipitation polymerization is shown in the SEM image of Figure 2a. The molar ratio of 1:4 for βCP; AA and 30 mL of acetonitrile favored the formation of nanoparticles [40]. The βCP–NP exhibited dimensions of regular shape and homogeneous distribution. Furthermore, DLS (Figure 2b) showed a mean diameter of 90.1 ± 5.6 nm with narrow size distribution. Indeed, the polydispersity index scored 0.23, where a value of less than 0.3 demonstrates that the size distribution is homogeneous [41]. The 1000 rpm speed of mechanical agitation was essential for forming βCP–NP with dimensions below 100 nm.

**Figure 3:**
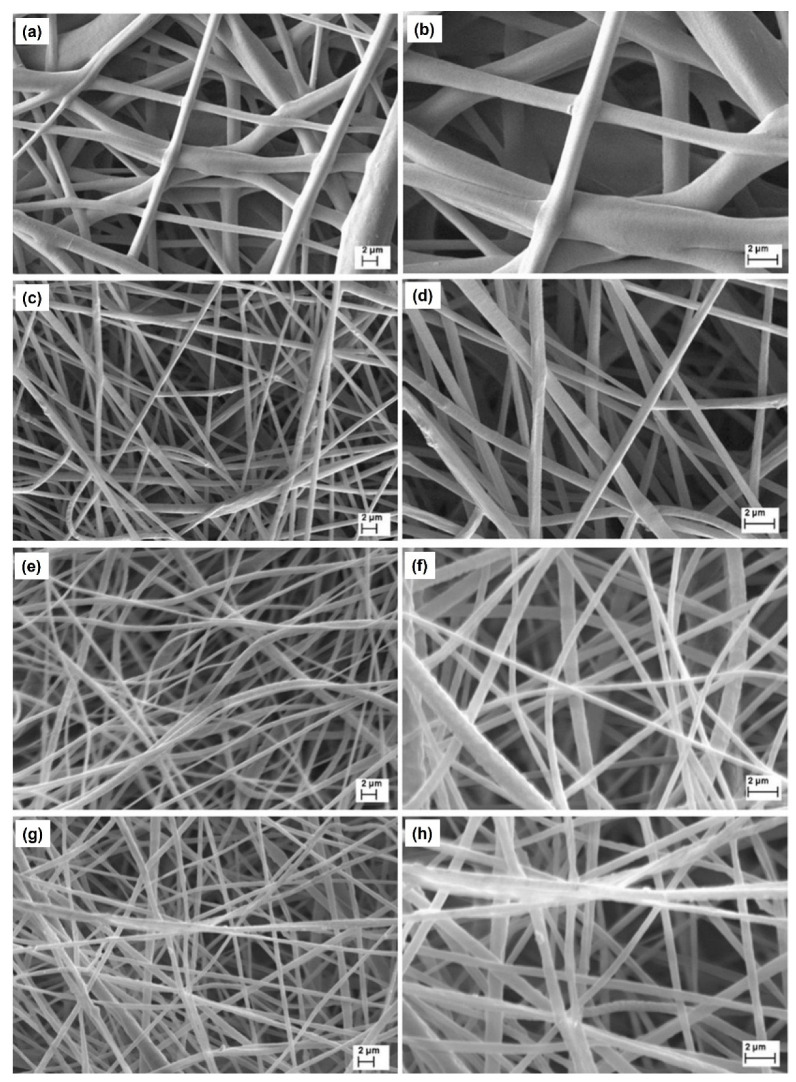
SEM images (5000× and 10,000× magnification) of PCL–fiber (**a**,**b**), MIM–βCP (**c**,**d**), functionalized MIM–βCP (**e**,**f**), and NIM (**g**,**h**).

Figure 3 shows SEM images of the PCL fibers. The PCL mat was produced with optimal processing conditions, such as stability of the Taylor cone, a wide area of deposition of the fibers, and absence of beads (Figure 3a,b). The images in Figure 3c,d show the morphology of MIM–βCP and reveal the absence of beads in its structure. The βCP–NP-loaded PCL fibers presented smaller diameters compared to the PCL fibers; the decrease was from 1.7 ± 1.1 µm to 0.7 ± 0.2 µm.

The MIM–βCP shown in Figure 3c,d was subjected to the functionalization process for removing βCP, which is a sequence of washing the MIM–βCP in methanol, as illustrated in Figure 1. Figure 3e,f shows the fiber morphology after washing, indicating that the functionalized MIM–βCP fibers remained intact with diameters of 0.7 ± 0.2 µm. Section 3.5 presents the analytical analysis of the washing rates from the functionalization process, showing the removal of βCP. As mentioned in Section 2.2, the NIM was fabricated for comparative study and was obtained with the same processing conditions as the MIM–βCP. Figure 3g,h shows the NIM morphology free of beads with diameters of 0.7 ± 0.2 µm.

In all cases of membrane processing, SEM showed qualitatively similar images for the electrospun fibers with or without βCP–NP. This morphology is typical of fibers collected with a rotary cylindrical collector with low rotation speed [42]. However, we aimed to characterize the surface roughness of the fibers to possibly identify differences among the samples, which we carried out by AFM, allowing direct 3D imaging with nanoscale resolution.

### 3.2. Three-Dimensional Nanoscale Morphological Surface Analysis

**Figure 4:**
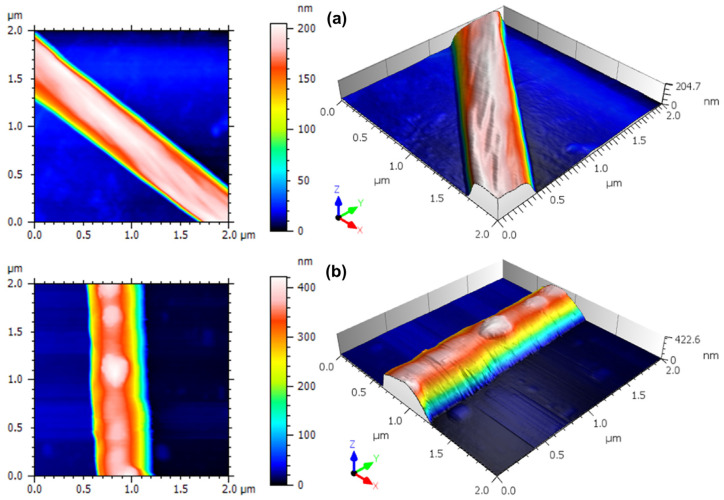
Top-view and 3-D rendering of large scope (2 × 2 µm^2^) representative AFM micrographs of the (**a**) PCL–fiber and (**b**) MIM–βCP, deposited over a glass substrate.

AFM is known for being a very sensitive morphological analysis technique that can evaluate subtle details in the spatial patterns of surfaces at the nanoscale [32,43,44,45,46,47]. In Figure 4, we report top-view and 3D rendering of representative AFM images obtained on a single PCL fiber without and with nanoparticles, respectively, at relatively large scope (2 × 2 µm^2^) that allows one to see a full transversal profile of the imaged fiber. The surface morphology of the PCL fiber presents the typical characteristics of electrospinning [48,49]. Liu et al. [50] observed a similar aspect when they fabricated fibers functionalized with poly (norepinephrine) to improve the hydrophilicity of PCL fibers. Moreover, a slight stretching along the fiber length appears, which is related to the fibrillar structure composed of both the amorphous and crystalline phase of the polymer [49].

**Figure 5:**
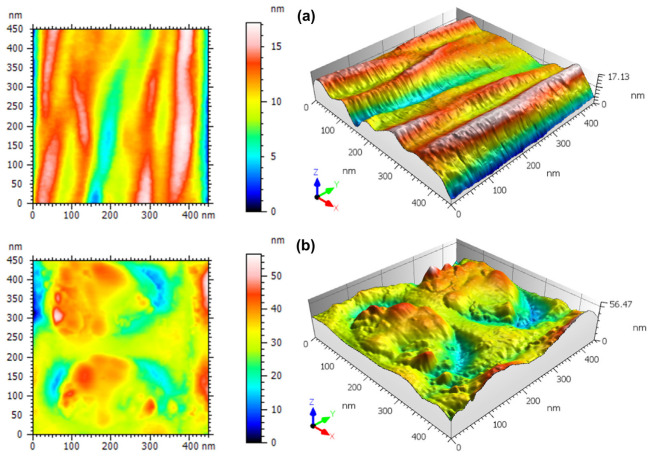
Top-view and 3-D rendering of high resolution (450 × 450 nm^2^) representative AFM micrographs of the (**a**) PCL–fiber and (**b**) MIM–βCP.

Figure 5 shows representative close-up (450 × 450 nm^2^) images of the surface morphology of single fibers, both PCL-only and βCP–NP-loaded PCL. In Figure 5a, the fiber-like nature of the surface is dominant, whereas in Figure 5b, the surface morphology appears to be almost isotropically rough, due to the presence of the βCP–NP. As one can see in Table 1, where the quantitative information extracted from these high-resolution images is reported, with the presence of the βCP–NP, some surface figures change considerably. The values of Sq, for example, changed from ~4 nm for the bare fiber to ~7 nm for the fiber with nanoparticles. In fact, this is the amplitude parameter most commonly used for describing the roughness, as it is the RMS of the height distribution, representing the spread of values around the single value of the best fitting plane.

The possible effect of the presence of nanoparticles on the PCL fiber surface could also be assessed by looking at the higher moments of the height distribution, namely, skewness Ssk and kurtosis Sku, which represent the distribution asymmetry and peakedness, respectively. Ssk is dimensionless as it is normalized by Sq. It is verified that there is an increase in the Ssk value, going from ~ −0.82 in the PCL to ~ −0.08 in the PCL + NPs. In both cases, however, asymmetry is negative (Ssk < 0), meaning that the surface is more porous-like rather than grain-like [51].

On the other hand, Sku is also a dimensionless yet positively defined parameter, for which three situations are possible: when its value is equal to 3, the surface has a Gaussian height distribution; for Sku < 3, the distribution is kind of flattened around the peak, whereas if Sk > 3 the region around the central peak is sharper. For the samples of PCL and PCL+ NPs, according to Table 1, Sku > 3, which is also called leptokurtic behavior [52]. This has sometimes been associated with a lower coefficient of static friction of the surface [53]. Not only is this property (Sku > 3) qualitatively similar for both samples, but also the respective values do not show a statistically significant difference between the samples.

In Table 1, we have reported three other height parameters, namely maximum peak height (Sp), maximum pit depth (Sv), and maximum height range (Sz = Sp + Sv). It was found that all showed the same tendency to increase values when there is the presence of the βCP–NP. Therefore, we can observe that the nanoparticles cause an increase in the height of the peaks.

### 3.3. Analysis of PCL–Fiber and MIM–βCP by XRD

**Figure 6:**
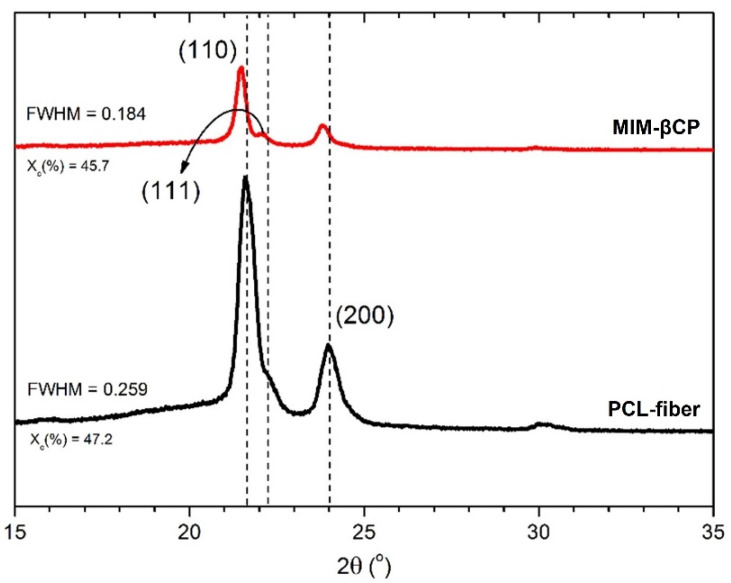
XRD patterns of PCL–fiber (black line) and MIM–βCP (red line).

Figure 6 shows the X-ray diffraction patterns for PCL–fiber and MIM–βCP. The PCL–fiber diffraction pattern (black curve) exhibited two intense peaks at diffraction angles of 21.6° and 23.9° which were assigned to the (110) and (200) reflection planes, respectively. The two peaks were associated with the orthorhombic crystal lattice of the PCL [55,56,57]. A shoulder was observed at a diffraction angle of 22.2° and was attributed at the (111) plane of the orthorhombic unit cell [58].

When compared to the MIM–βCP (red curve), a similar X-ray diffraction pattern profile was observed, with peaks of lower intensity of the diffraction angles in relation to the fiber–PCL diffraction pattern. The X-ray diffraction angles were assigned at 21.4° and 23.7°, respectively. A left-shift of 0.2° was observed, suggesting that the PCL–fiber crystal lattice increased its spacing to accommodate the βCP–NP. From the area of the diffraction pattern curve, the fiber–PCL showed a crystallinity (Xc) of 47.2% and an amorphous phase of 52.8%, while the MIM-BCP showed a crystallinity reduced to 45.7% and an amorphous phase increased to 54.3% due to the presence of βCP–NP.

Monteiro et al. showed that the crystallization of the PCL was affected with the introduction of nanoparticles and a specific drug, modifying its semicrystalline structure of the PCL and reducing its crystallinity [59]. After the addition of βCP–NP, there was a decrease in full width at half maximum (FWHM) the peak of the plane (110), corresponding to a narrowing of the peak by 21.4°. Generally, the narrowing of a crystalline peak can be attributed to the increase in size of the crystal or to the reduction in the structural disturbance in the sample; this may indicate that the presence of the βCP–NP affected the shape of the crystal without changing the orthorhombic crystalline phase of the PCL yet reflecting in a more rugged morphology.

### 3.4. Calibration Curves

**Figure 7:**
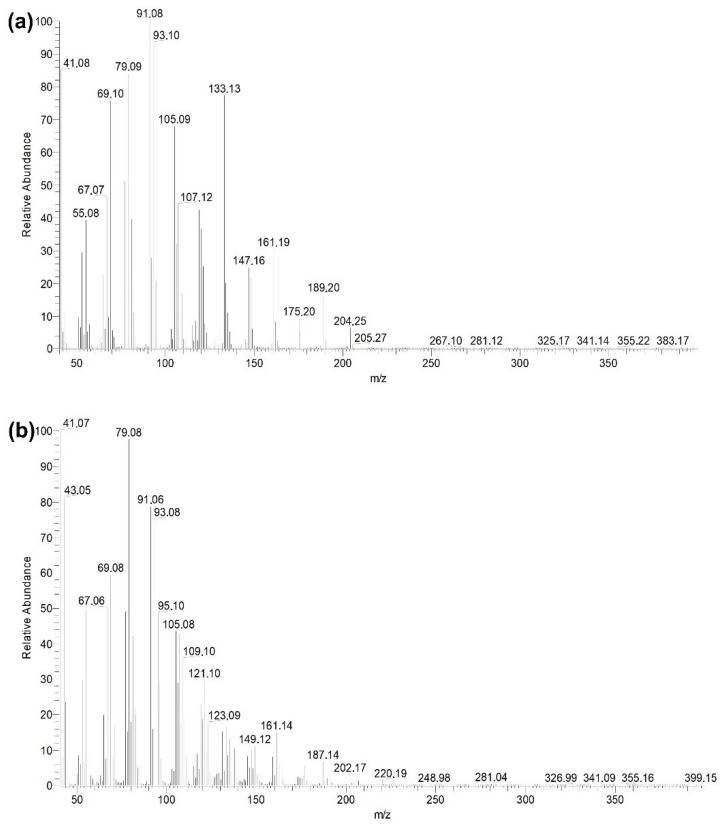
Mass spectrum of βCP (**a**) and of CP–oxide (**b**) obtained by GC-MS.

Figure 7 shows the chromatographic profiles of βCP and CP–oxide obtained by GC-MS, where the retention times were 9.48 and 12.96 min. The mass spectra show the βCP and CP–oxide retention times shown in Figure 7a,b, respectively [39,60]. The calibration curves for the βCP and CP–oxide were y = 2.0 × 10^9^ [C]+3.0 × 10^8^ with a correlation coefficient R^2^ = 0.9940 and y = 1.4 × 109 [C]−2.1 × 108 with R^2^ = 0.9920, respectively.

### 3.5. Functionalization of the MIM–βCP

**Figure 8:**
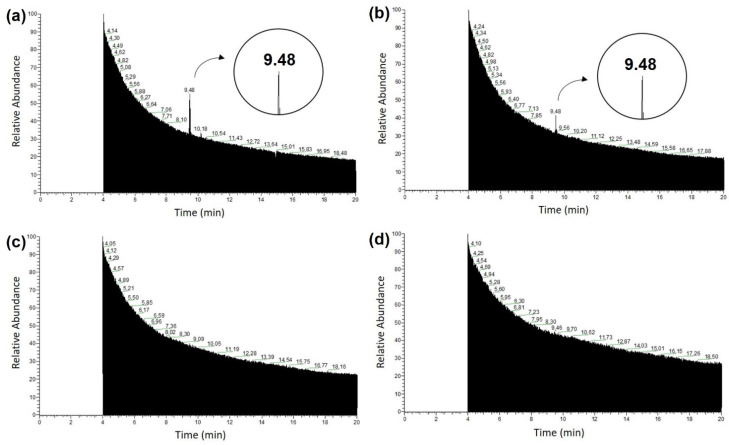
Chromatographic profiles of the functionalization process of MIM–βCP by washes in methanol. (**a**) First wash, (**b**) second wash, (**c**) third wash, and (**d**) fourth wash. The retention time of 9.48 min indicates the removal of the βCP from MIM–βCP.

The chromatographic profiles of the functionalization process of MIM–βCP are shown in Figure 8. In Figure 8a, an intense peak in retention time attributed to βCP at 9.48 min is shown, which represents its removal from the MIM–βCP structure during the first wash. During the second wash, Figure 8b shows a peak in retention time that is less intense than the peak in Figure 8a. Figure 8c,d shows the absence of the peak during the third and fourth washes, demonstrating the total removal of the βCP from the MIM–βCP.

After the functionalization process, the functionalized MIM–βCP was characterized by SEM. This functionalization process preserved the morphology of the fibers, as shown in Figure 3e,f.

### 3.6. βCP Extraction and Binding Capacity

**Figure 9:**
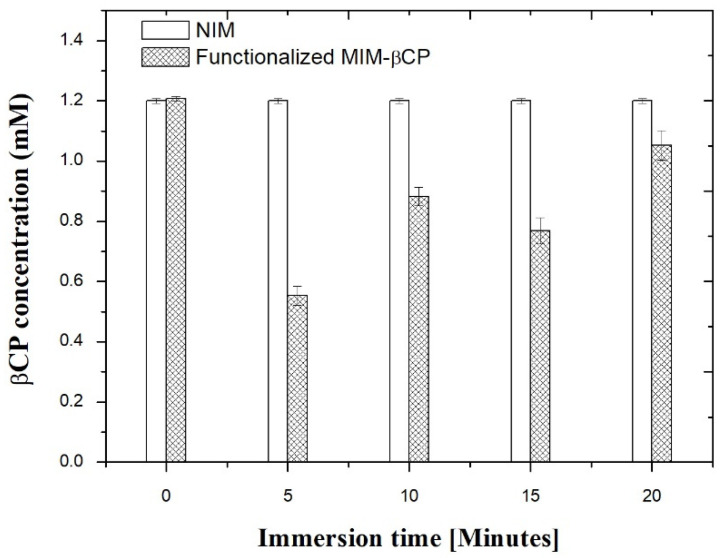
βCP concentration versus immersion time at 5 min intervals using the functionalized MIM–βCP and NIM.

Figure 9 shows the graph of βCP concentration versus immersion time of the functionalized MIM–βCP in a 1.2 mM βCP solution. This study was carried out in triplicate with time intervals of 5 min. The values of the unknown concentrations were calculated using the βCP calibration curve (y = 2.0 × 10^9^ [C] +3.0 × 10^8^) from the peak area obtained in each chromatographic profile of aliquots collected at the times 0, 5, 10, 15, and 20 min.

After 5 min of immersion, the adsorption effect of the functionalized MIM–βCP into βCP solution reduced the 1.2 mM concentration to 0.55 ± 0.03 mM, equivalent to the extraction of 54.4 ± 2.4% βCP from the βCP solution. The adsorption effect for 10 min of immersion was the βCP extraction of 27.1 ± 2.3%; this value is minor compared to that at the 5-min immersion time. The βCP extractions at 15 and 20 min of immersion were 36.6 ± 3.4 and 13.1 ± 4.6%, respectively. The NIM did not show an adsorption effect into the 1.2 mM βCP solution. The functionalized MIM–βCP showed rapid adsorption for βCP, which may be related to its porous structure. On the other hand, the βCP extraction was less after the immersion time of 5 min. This may have occurred due to mechanical agitation during the experiment that disturbed the adsorption equilibrium. However, even so, the time of 5 min was used to calculate the binding capacity of the functionalized MIM–βCP and NIM, using Equation (4) based on the methodology used by Scorrano et al. [61]. The values were Q_MIM_ = 1.80 ± 0.05 µmol/cm^2^ and Q_NIM_ = 0.30 ± 0.03 µmol/cm^2^, respectively.

### 3.7. Selectivity Test

**Figure 10:**
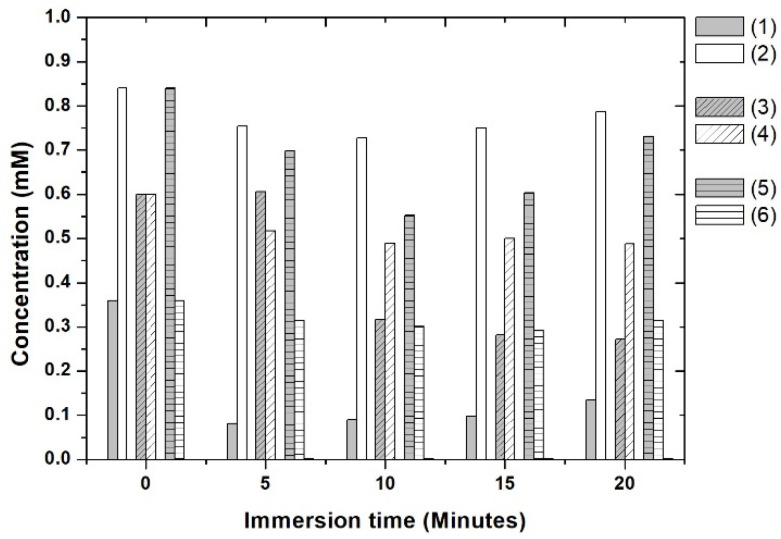
Concentrations of the binary solutions of βCP (1, 3, and 5) and CP–oxide (2, 4, and 6) from known concentrations in different ratios of 30/70, 50/50, and 70/30, respectively.

A selectivity test was performed with binary solutions of βCP/CP–oxide of 30/70, 50/50, and 70/30 ratios, respectively. The chromatographic profiles were obtained by GC-MS, and calibration curves (βCP and CP–oxide) were used. In Figure 10 and Table 2, the results of the selectivity test are presented.

In the immersion time of 5 min, MIM–βCP was more efficient in extracting βCP (77%) than CP–oxide (10%). At the times of 10, 15, and 20 min, the βCP extraction efficiency gradually decreased. The functionalized MIM–βCP showed higher values of βCP extraction efficiency in the 30/70 binary solution.

It was observed that the extraction efficiency values of the 50/50 and 70/30 binary solutions decreased as the concentration of βCP increased, indicating that the selectivity of functionalized MIM–βCP is more efficient at lower concentrations of βCP. Furthermore, although the CP–oxide molecule presents a structural formula like βCP, the functionalized MIM–βCP showed excellent molecular recognition capacity for βCP.

## 4. Conclusions

MIM for βCP extraction was successfully produced by electrospinning. Furthermore, we present the synthesis of βCP–NP. SEM and AFM images showed morphological changes after the βCP–NP incorporation into PCL–fibers that may favor the adsorption of βCP in solution. The XRD investigation showed an increase in crystal lattice spacing and a decrease in crystallinity, contributing to the accommodation of βCP–NP in the polymer matrix. The selectivity test confirmed the preferential adsorption of βCP on functionalized MIM–βCP, despite the presence of an interferent in different proportions (CP–oxide), proving the ability to recognize functionalized MIM–βCP. Electrospun MIM–βCP is an innovative material that has potential in versatile applications, such as tissue engineering as a treatment for skin diseases, in filters for extracting and detecting βCP to prevent counterfeiting of commercial βCP-based products, and in smart clothing with the property of insect repellency.

## Figures and Tables

**Table 1:** Height distribution parameters obtained from the analysis of representative AFM images of the samples, according to ISO 25178-2:2012 [54].

Parameter	Unit	PCL	PCL + NP
Sq	[nm]	4.1 ± 0.7	7.2 ± 1.8
Ssk	[-]	−0.8 ± 0.3	−0.1 ± 0.1
Sku *	[-]	5.0 ± 1.7	4.6 ± 1.3
Sp	[nm]	8.9 ± 2.6	26.2 ± 5.1
Sv	[nm]	21.4 ± 8.9	41.1 ± 7.5
Sz	[nm]	30.3 ± 11.4	67.3 ± 10.0

* Samples without statistically significant difference (*p* < 0.05).

**Table 2:** Extraction of βCP and CP–oxide from binary solutions of ratios 30/70, 50/50, and 70/30.

Time	Binary Solution (30/70)		Binary Solution (50/50)		Binary Solution (70/30)	
Min.	βCP	CP–Oxide	βCP	CP–Oxide	βCP	CP–Oxide
0	0	0	0	0	0	0
5	77%	10%	0	14%	17%	13%
10	75%	13%	47%	18%	34%	16%
15	73%	10%	53%	17%	28%	19%
20	63%	6%	55%	19%	13%	13%

## Data Availability

Not applicable.
